# Professor Wawruch of Vienna on the Tape Worm

**Published:** 1842-04-23

**Authors:** 

**Affiliations:** Vienna


*Remarks on the Tape-Worm.* By Professor WAWRUCH of Vienna.—Professor Wawruch has, during the last twenty years, had an opportunity of treating no fewer than 206 cases of persons affected with tape-worm, and as it falls to the lot of very few indeed to see anything like this number of cases, his observations on the causes, symptoms, and treatment are highly valuable.

He remarks that 71 cases occurred in males, and 135 in females. 22 were below 15 years of age, one only $3\frac{1}{2}$; but the disease was most frequent in those from 15 to 40 years of age. In one only the age was 54. The disease occurred chiefly among the lower classes residing on the banks of the Danube, in low damp habitations, and the causes which favoured the production of scrofula seemed also to give a predisposition to tape-worm.

Gastric and cutaneous affections, but especially gastric and intermittent fevers, seemed to precede the appearance of tenia in most cases; but other diseases, such as chlorosis, syphilis, scurvy, which are remarked to have a weaning influence on the digestive organs, were also observed to be followed by tape-worm. Almost all those who had tape-worm were subject to lumbrici in their youth. He does not think this is a hereditary disease; two instances alone having come under his notice, of parent and child being at the same time affected with the disease.

The diet which seemed especially to favour the generation of tape-worm was bad bread, farinaceous articles of food, milk, butter, cheese, potatoes, pork, mutton, and bad water.

In most cases the females affected with tape-worm had their menstrual secretions disordered, and were suffering in consequence.

The duration of the disease was from a few months to many years; cases having come under his notice where the disease lasted from ten to thirty-five years.

The *Botriocephalus* occurred in only three cases out of the 206 cases of tape-worm; the *Tænia solium* in all the rest.

The symptoms which attended the presence of tape-worm were very various, but those which appeared most constantly were the following:—Dull pain in the forehead, dizziness, and ringing in the ears; indistinct and troubled vision, in one case periodic day-blindness; eyes surrounded with a dark or blue circle; œdema of the upper eyelid; dilated pupils, frequent involuntary spasms of the eyes. The colour of the countenance often changed; the lips were ash-coloured, and a particular appearance existed around the mouth and nose; sometimes they had a cachectic appearance, at other times they appeared to be in robust health. Anorexy, alternating with an appetite so voracious that if they did not get somewhat to satisfy the unnatural craving for food they almost fainted; cravings for particular kinds of food were also remarked. Fetid breath, a loaded tongue, salivation, and efforts to vomit, with pruritis of the nose, anus, &c., grinding of the teeth, palpitations, and general uneasiness were frequent symptoms. The belly was swollen, and a gurling sound was heard; there was present a feeling of constriction or pricking around the umbilicus; and the undulatory movements of a foreign body were felt in the intestines in the morning; but these sensations were relieved by the ingestion of food, or by drinking warm coffee and milk.

When the disease has lasted for a long time, and the persons are of an irritable constitution, a train of nervous symptoms make their appearance, as melancholia, lipothyrnia, frightful dreams, general or partial convulsions, St. Vitus's dance, epilepsy, aphonia, &c. But the most certain pathognomonic sign of the presence of tænia is the passing of one or more joints of the animal, which occurs spontaneously or after severe attacks of disease, as scarlatina, typhoid fevers, &c., or after the use of certain articles of food, as cucumbers, garlic, raddish, &c. The joints of the tænia are most generally thrown off at a determinate period, for the most part at the waning of the moon, or at the new moon, at which time there is an exacerbation of all the symptoms. The joints of the tape-worm are either passed alone, or along with the stools; they generaly exhibit signs of life, and the larger the number of joints the longer do they continue to live.

The whole 206 cases were treated alike; but no cure was ever attempted, so long as there existed a decidedly cachectic state, chronic inflammation of the intestines, chronic diarrhœa, hæmorrhoides, or pregnancy; and the treatment was always deferred in females till six days after the menstrual period. The patients were subjected to a preparatory treatment which Professor Wawruch considered indispensable in all. He therefore gave them daily for three or four days a simple demulcent decoction, with sal ammoniac, and confined their diet to beef-tea three times a day. In eight cases this rigid diet sufficed to expel the tape-worm. The evening before the proper treatment commenced, he administered to his patient a dose of fat soup without salt, and after that an enema, which was repeated the next day, but not immediately after the soup or the anthelmintics. These consist of two ounces of castor oil, which is taken during the day in divided doses, alternated with the powdered root of the male fern, in the dose of from fifteen to thirty grains. To aid the passage of the worm into the large intestines, an enema with oil or milk was administered after every dose of oil and fern-root. The tape-worm was always more sure to be expelled when a drastic purgative composed of equal parts of calomel, gamboge, and sugar, two to eight grains of each, was given. It was frequently necessary to repeat this dose from three to six times before the worm was expelled. In a few cases strong doses of the drastic purgative had no effect in evacuating the bowels, in which case the sulphate of potash generally succeeded.

When the tape-worm was expelled in a mass, the whole animal appeared to come away, as no medicines afterwards administered brought away even a single joint; and in all those who had taken the male-fern powder, the worm was found torn in pieces, even the single joints were broken up. The periods at which the worm was expelled varied. Thus eight were expelled in consequence of the preparatory regimen; 13 by the anthelmintics; 11 by the first dose of the drastic purgative; 14 by the second dose; and 15 by the third, and, in general, between the first and second hour after the administration of the drastic purgative. In some cases it was the second, third, and fourth day before the worm was expelled, and in one only it was the twelfth day. The morbid symptoms disappeared so rapidly after the expulsion of the tape-worm, that the patients generally quitted the hospital the second day thereafter.

Of the 206 patients, in twenty the complaint returned a second time; in five, a third time; and in one, a fourth time, at periods of time varying from one month to two years. Of the 206 patients, 151 were cured; 17 were not subjected to any treatment; the occurrence of unfavorable symptoms caused the treatment to be suddenly stopped in 19; in 13 the remedies were administered during full moon and failed; and in 6 there existed lumbrici. It is mentioned as a curious fact, that when lumbrici existed in the intestinal tube, the treatment had no effect on the tæniæ, but expelled the lumbrici alone.—*Ed. Med. and Surg. Journ., from Med. Jahr. des Oester. Staates.* February, 1841.

